# Obinutuzumab in the treatment of idiopathic refractory membranous nephropathy: an observational case series

**DOI:** 10.3389/fmed.2026.1704970

**Published:** 2026-02-11

**Authors:** Xiangling Zhao, Xuejie Chang, Yuejuan Wang, Duqun Chen, Manshu Yu, Jing Zhao, Hong Zhu, Yan Mao, Chenxia Juan

**Affiliations:** 1 Jiangsu Province Hospital of Chinese Medicine, Affiliated Hospital of Nanjing University of Traditional Chinese Medicine, Nanjing, China; 2 National Clinical Research Center for Kidney Diseases, Jinling Hospital, Affiliated Hospital of Medical School, Nanjing University, Nanjing, China

**Keywords:** anti-PLA2R antibodies, CD20 B cells +, idiopathic refractory membranous nephropathy, obinutuzumab, PLA2R antibody

## Abstract

**Objective:**

Idiopathic refractory membranous nephropathy (IRMN) is a subset of nephrotic syndrome with a high risk of progression to end-stage renal disease, yet treatment options remain limited. Obinutuzumab, a humanized type II anti-CD20 antibody, induces efficient CD20+ cell depletion, leading to favorable immunological and proteinuria responses in IRMN. This study sought to assess the effectiveness and safety of obinutuzumab for treating IRMN.

**Methods:**

A total of 31 patients with IRMN were enrolled and received obinutuzumab therapy. They were followed for 12 months. The primary endpoint was the remission rate (complete or partial remission). Secondary outcomes included changes in proteinuria, serum albumin, serum creatinine, anti-PLA2R antibody levels, CD20/CD19 cell counts, and adverse events.

**Results:**

At 12 months, the overall remission rate was 80%, with 16% achieving complete remission and 64% achieving partial remission. The median time to remission was 4.5 months (IQR 2.0–7.0 months). The immunological remission rates at 3, 6, 9, and 12 months were 70, 83, 90, and 93%, respectively. Regarding anti-PLA2R antibodies, 80% of patients showed a rapid decline within the first 3 months (*p* < 0.001), with 64% achieving a > 50% reduction from baseline by month 3. At the last follow-up, all patients had achieved seroconversion to anti-PLA2R antibody negativity. Adverse events included infusion-related reactions such as fever, hypotension, tachycardia, and flushing. No deaths occurred.

**Conclusion:**

Obinutuzumab effectively induces remission in patients with IRMN and demonstrates a tolerable safety profile.

## 1 Introduction

Membranous nephropathy (MN) is a common glomerular disease, with a reported global annual incidence of approximately 1.2 per 100,000 person-years (1). It occurs more frequently in males. Nearly 60% of patients diagnosed with MN present with nephrotic syndrome, characterized by proteinuria, hypoalbuminemia, hyperlipidemia, and edema, often accompanied by hypertension and hematuria, while others exhibit only asymptomatic proteinuria (2). Idiopathic membranous nephropathy (IMN) constitutes the majority of adult MN cases (3). Growing evidence supports a pivotal role of autoimmunity in its pathogenesis (4). Specific biomarkers, notably anti-PLA2R antibodies, are detectable in 70–80% of IMN patients via renal biopsy, with increased glomerular PLA2R antigen staining showing synchrony with the presence of serum autoantibodies (5). Anti-PLA2R antibodies are autoantibodies generated against podocyte antigens. Studies have demonstrated their high sensitivity in differentiating IMN (6, 7). Furthermore, anti-PLA2R antibody titers are associated with disease activity, severity, and prognosis (8, 9). Notably, nearly one-third of anti-PLA2R antibody-positive IMN patients eventually progress to chronic kidney disease (8, 10).

According to the 2021 Kidney Disease: Improving Global Outcomes (KDIGO) guidelines, the first-line therapeutic options for idiopathic membranous nephropathy (IMN) include rituximab and cyclophosphamide in combination with corticosteroids, among other immunosuppressive agents (11). Among these regimens, anti-CD20 therapy, exemplified by rituximab, is firmly grounded in a well-defined pathophysiological basis. Previous studies indicate that autoimmune injury mediated by CD20+ B cells represents a core pathological mechanism in IMN (12). Anti-CD20 therapy targets and depletes precursor B cells responsible for producing anti-PLA2R antibodies, thereby inhibiting the generation of autoantibodies. This approach is particularly beneficial for patients with high anti-PLA2R antibody titers (13). However, the efficacy of rituximab is constrained by limitations such as delayed response, drug resistance, premature B-cell reconstitution, and renal excretion (14, 15), which collectively lead to suboptimal clinical outcomes. Consequently, the reported overall remission rate for rituximab in IMN patients is approximately 65% (16). As a result, a considerable proportion of patients fail to achieve adequate disease control and progress to idiopathic refractory membranous nephropathy (IRMN). According to the KDIGO 2021 Clinical Practice Guideline and expert consensus (11, 17, 18), these patients are defined as those with IMN who fail to achieve clinical remission—defined as a > 50% reduction in proteinuria and a 24-h urinary protein excretion < 3.5 g—after at least 6 months of adequate and conventional immunosuppressive therapy. Patients with IRMN have a poor prognosis and constitute a group at high risk of progressing to end-stage renal disease (19). Therefore, developing novel and effective treatments for IRMN remains an urgent clinical priority in nephrology.

Obinutuzumab, a next-generation anti-CD20 monoclonal antibody developed following rituximab, features a fully humanized structure (20). Compared to rituximab, which also targets CD20, obinutuzumab induces more effective depletion of CD20+ B cells. Pharmacokinetic studies have demonstrated that obinutuzumab achieves a significantly higher receptor occupancy required for half-maximal response than rituximab (21). As a result, obinutuzumab serves as an important salvage therapy for patients with membranous nephropathy who previously showed inadequate response to rituximab or other conventional immunosuppressive treatments (22). Currently, there are limited studies on the use of obinutuzumab in idiopathic refractory membranous nephropathy (IRMN), and available results are inconsistent, failing to provide a clear assessment of its true therapeutic efficacy. To address this gap, we conducted a study to evaluate the clinical efficacy and safety of obinutuzumab in the treatment of IRMN.

## 2 Materials and methods

### 2.1 Case collection

This study enrolled 31 patients with idiopathic refractory membranous nephropathy (IRMN) who were treated with obinutuzumab at the Department of Nephrology, Jiangsu Provincial Hospital of Traditional Chinese Medicine between April 2023 and March 2025. Clinical data were systematically collected and archived. The inclusion criteria were as follows: (i) Age of 18 years or older. (ii) A confirmed diagnosis of idiopathic membranous nephropathy based on KDIGO criteria. (iii) Evidence of treatment-resistant disease, manifested as persistent 24-h urinary protein ≥ 3.5 g or a < 50% reduction from baseline, after a minimum of 6 months of therapy with protocols involving corticosteroids and other immunosuppressants (e.g., cyclophosphamide, calcineurin inhibitors, or rituximab). (iv) No prior exposure to obinutuzumab. (v) Availability of comprehensive clinical data and a documented follow-up period of at least 12 months. Exclusion criteria comprised: (i) secondary membranous nephropathy; (ii) non-adherence to the study protocol; and (iii) irregular follow-up or loss to follow-up. The study protocol was approved by the Institutional Ethics Committee (Approval No: 2025NL-092-02), and written informed consent was obtained from all participants after detailed consultation.

### 2.2 Definitions

Clinical remission was categorized as either complete or partial, according to the Kidney Disease: Improving Global Outcomes (KDIGO) guidelines (11). Complete remission (CR) was defined as 24-h urinary protein excretion < 0.3 g/24 h, accompanied by serum albumin ≥ 35 g/L and stable renal function. Partial remission (PR) was defined as urinary protein < 3.5 g/24 h with a > 50% reduction from baseline, serum albumin ≥ 30 g/L, and stable renal function. Disease relapse was defined as a recurrence of proteinuria > 3.5 g/24 h after achieving remission. B-cell depletion was classified as CD20^+^ cells < 5 cells/μL, complete B-cell depletion as < 1 cell/μL, and B-cell reconstitution as CD20^+^ cells ≥ 5 cells/μL (23). Immunological remission was defined as an anti-PLA2R antibody (aPLA2Rab) level < 14 RU/mL, with complete immunological remission also set at < 14 RU/mL. Risk stratification was performed based on KDIGO guidelines and expert consensus (24). High-risk disease was defined as: urinary protein > 8 g/24 h persisting for over 6 months; urinary protein > 5 g/24 h after 3 months of supportive care, or > 3.5 g/24 h after 6 months with < 50% reduction from baseline, plus at least one of the following: (1) serum albumin < 25 g/L, or (2) aPLA2Rab ≥ 150 RU/mL. Refractory membranous nephropathy was defined as persistent proteinuria ≥ 3.5 g/24 h after at least 6 months of immunosuppressive therapy (mono- or combination therapy), with or without progressive renal impairment during treatment (19).

The primary endpoint was the remission rate, comprising both complete remission (CR) and partial remission (PR). Secondary endpoints included changes in urinary total protein (UTP), serum albumin (ALB), serum creatinine (Scr), anti-PLA2R antibody titers, and CD20^+^ cell counts, which served as efficacy indicators for obinutuzumab treatment. Safety was evaluated based on the incidence of adverse events.

### 2.3 Statistical analysis

Data analysis was performed using SPSS version 26.0 and GraphPad Prism software. Descriptive statistics were applied for qualitative variables, including demographic characteristics and comorbidities such as hypertension and hyperlipidemia, which were summarized as frequencies, percentages, or proportions. Normally distributed continuous variables were expressed as mean ± standard deviation and compared between groups using the independent samples *t*-test. Non-normally distributed data were presented as median (25th–75th percentiles) and analyzed with non-parametric tests; the Mann-Whitney U test was employed for comparisons between two groups. For comparisons involving two groups, if assumptions of normality and homogeneity of variance were met, an independent *t*-test was used; otherwise, the Mann-Whitney U test was applied. A two-sided *p*-value < 0.05 was considered statistically significant. Time-to-event outcomes, including remission and complete remission, were analyzed using the Kaplan–Meier method, and between-group differences were compared with the log-rank test.

### 2.4 Materials and reagents

Obinutuzumab (Gazyva^®^) was provided by Roche Diagnostics GmbH under clinical trial supply agreement. Obinutuzumab was diluted in 1,000 mL normal saline. Obinutuzumab was administered as a 3.0 g cumulative dose per treatment cycle, given as three separate 1.0 g infusions at weeks 0, 2, and 24. A standard premedication regimen was administered 24 h before each obinutuzumab infusion, comprising intravenous methylprednisolone (80 mg) and oral acetaminophen (1,000 mg), with an additional intravenous dose of dexamethasone (20 mg) given on the infusion day. Any concurrent immunosuppressive agents (e.g., corticosteroids, cyclophosphamide, tacrolimus) were withdrawn immediately prior to obinutuzumab initiation. Prior to enrollment, 14 patients (45%) had already received optimal RAAS inhibitor therapy. For these patients, in whom contraindications and intolerance had been excluded, RAAS inhibitors were maintained throughout the obinutuzumab treatment period.

The study was approved by the Institutional Ethics Committee at the study site and was conducted in accordance with applicable regulatory requirements [Ethics Approval No.: (2025NL-092-02)], the principles of the Declaration of Helsinki, and the International Council for Harmonization Good Clinical Practice guidelines. Written informed consent was obtained from all participating patients.

## 3 Results

### 3.1 Baseline

A total of 31 patients with idiopathic refractory membranous nephropathy were included in the final analysis for clinical efficacy evaluation. The cohort consisted of 25 males (80%) and 6 females (20%), with a mean age of 48.8 ± 12.5 years. Key baseline clinical characteristics and prior treatment histories are summarized in Table 1.

**TABLE 1:** Baseline characteristics of the study cohort.

Characteristic	Total (*n* = 31)
Sex, male/female, n (%)	25 (80) /6 (20)
Age, years	48.8 ± 12.5
Age by tertile, n (%) ( < 40/40–50/ > 50 years)	9/5/17
Disease duration, months	33 ± 30
24-h urine protein, g/24 h	7.1 ± 3.5
Serum albumin, g/L	27.5 ± 4.7
Serum creatinine, μmoL/L	100.2 ± 58.7
Anti-PLA2R antibody titer, RU/mL	141.5(77.2,261.2)
Comorbidities hypertension, n(%)	20 (62)
Comorbidities hyperlipidemia, n(%)	13 (40)
Comorbidities hyperuricemia, n(%)	8 (25)
Comorbidities diabetes, n(%)	9 (28)
**Previous treatments before obinutuzumab, n**	
ACEI/ARB	14
Glucocorticoids and cyclophosphamide	19
Calcineurin inhibitors	16
Rituximab	10

A total of 31 patients were evaluated from baseline through 24 weeks of obinutuzumab treatment. None of the patients had achieved remission (defined as urinary protein excretion < 0.3 g/24 h) prior to obinutuzumab initiation. Only three patients exhibited urinary protein levels between 0.15 and 3.5 g/24 h, while the remaining 28 patients (88%) presented with severe proteinuria, with a mean baseline value of 7.1 ± 3.5 g/24 h. All patients were positive for anti-PLA2R antibodies at baseline. Among them, 23 patients (71%) had undergone renal biopsy, all of whom showed positive anti-PLA2R staining in renal tissue. The median anti-PLA2R antibody titer was 141.5 RU/mL. Mean serum creatinine and serum albumin levels were 100.2 ± 58.7 μmoL/L and 27.5 ± 4.7 g/L, respectively. Before obinutuzumab therapy, 19, 16, and 10 patients had been previously treated with glucocorticoids plus cyclophosphamide, calcineurin inhibitors, and rituximab for more than 6 months, respectively. Sixteen patients had received two or more lines of combination therapy. Despite this, none of the patients attained controlled proteinuria levels. The majority of patients also had underlying comorbidities such as hypertension, hyperlipidemia, and diabetes.

### 3.2 Clinical efficacy and treatment responses

**FIGURE 1:**
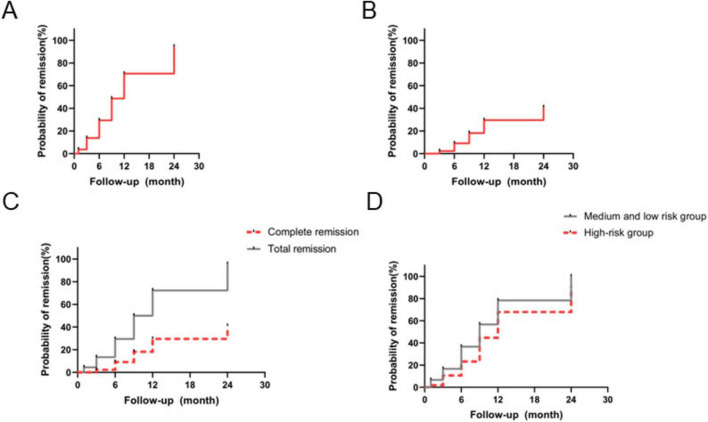
Clinical efficacy. **(A)** Overall response rate (ORR) in all patients; **(B)** complete response rate (CR) in all patients; **(C)** ORR versus CR in the study cohort; **(D)** comparison of ORR and CR between high-risk and intermediate/low-risk groups.

Clinical remission rates for all patients are summarized in Figures 1A–C. At 12 months after obinutuzumab treatment, 25 patients achieved either complete or partial remission, corresponding to an overall remission rate of 80%. Specifically, 5 patients (16%) attained complete remission and 20 (64%) achieved partial remission. The median time to remission for the entire cohort was 4.5 months (IQR 2.0–7.0 months). Based on risk stratification, patients were categorized into intermediate-low risk (*n* = 13, 42%) and high-risk (*n* = 18, 58%) groups. Comparative analysis of remission rates between these groups is shown in Figure 1D. The intermediate-low risk group reached a near 99% remission rate by month 9, achieving 100% remission by month 12, including 4 cases of complete remission. In contrast, the high-risk group exhibited a remission rate of 72% at 12 months, comprising 12 partial remissions, 1 complete remission, and 1 relapse. The remission rate was significantly higher in the intermediate-low risk group compared to the high-risk group (*p* = 0.03), with a hazard ratio (HR) of 0.73 (95% CI 0.50–1.07).

### 3.3 Changes in clinical parameters

**FIGURE 2:**
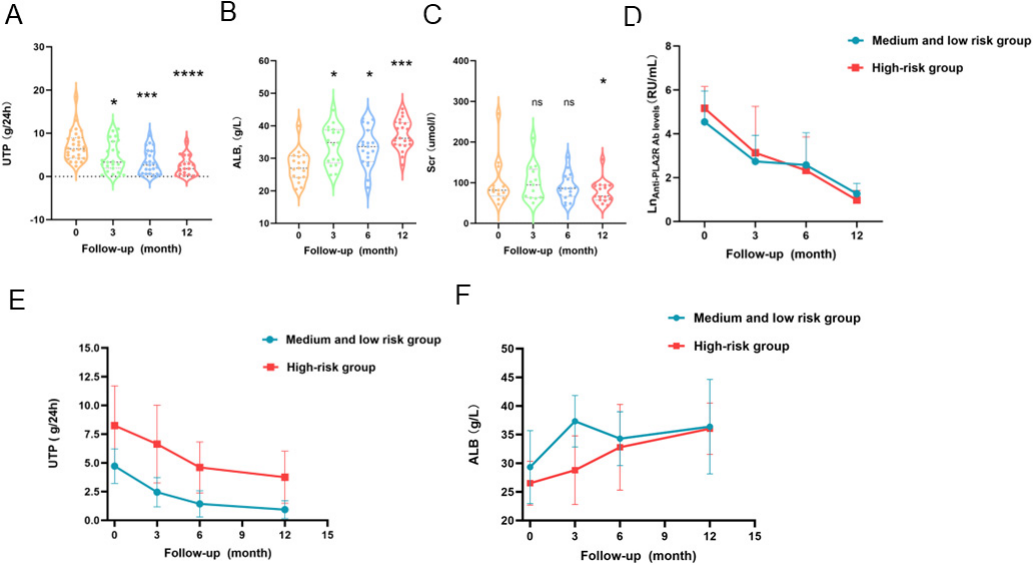
Change trend graph of efficacy indicators. **(A)** 24-h urinary protein (UTP) changes over time (0, 3, 6, 12 months) in all patients; **(B)** serum albumin (ALB) recovery over time in all patients; **(C)** serum creatinine (Scr) evolution over time. Subgroup analysis: Trend comparison of anti-PLA2R antibody titers **(D)**, UTP **(E)**, and ALB **(F)** between high-risk and low-risk groups over time. UTP, 24-h urinary protein excretion; ALB, serum albumin; Scr, serum creatinine. (*p < 0.05, ^**^p < 0.01, ^***^p < 0.001, ^****^p < 0.0001).

The effects of obinutuzumab treatment on urinary total protein (UTP), serum albumin (ALB), and serum creatinine (Scr) are shown in Figure 2. A significant downward trend in UTP was observed. Compared to the baseline level of 7.1 ± 3.5 g/24 h, UTP decreased steadily after the first infusion and reached 2.3 ± 2.2 g/24 h at the final follow-up (*p* < 0.0001). The mean percentage reduction in proteinuria was 42.3% at 3 months. By month 12, half of the patients had achieved a reduction exceeding 50%, with a mean reduction of 74.8% among responders at the last follow-up. When stratified by risk, both the intermediate-low and high-risk groups showed similar declining trends in UTP over time. However, the intermediate-low risk group exhibited a more rapid decline within the first 3 months and maintained significantly lower UTP levels throughout the treatment period (*p* < 0.05), indicating a higher response rate to obinutuzumab in this subgroup. Serum albumin levels increased significantly from a baseline of 27.5 ± 4.7 g/L to 34.1 ± 5.8 g/L at the end of follow-up (*p* < 0.05). Although the high-risk group demonstrated a more gradual increase, the intermediate-low risk group showed a steeper rise within the first 3 months (*p* < 0.05). After month 3, albumin levels in the intermediate-low risk group fluctuated moderately, and no significant difference was observed between the two groups by month 12. Serum creatinine decreased from 100.2 ± 58.7 μmoL/L at baseline to 76.9 ± 28.2 μmoL/L after the last administration. Although no significant difference was found between the 12-month value and baseline (*p* = 0.438), a downward trend was noted in patients with elevated baseline Scr levels.

### 3.4 Anti-PLA2R antibody dynamics and immunological remission

**FIGURE 3:**
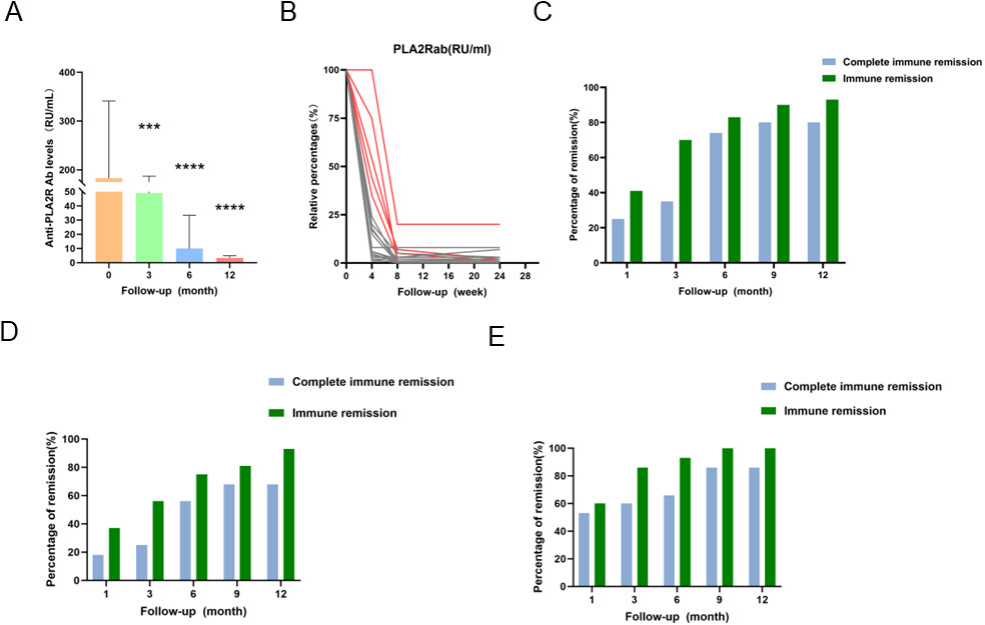
Anti-PLA2R antibody dynamics and immunological remission. **(A)** Bar plot showing longitudinal changes in anti-PLA2R antibody titers for all patients. **(B)** Line graph depicting relative percentage change in anti-PLA2R levels (baseline set as 100%). **(C)** Immunological complete/partial remission rates in the overall cohort. **(D)** Immunological remission rates in patients with baseline aPLA2Rab > 100 RU/mL. **(E)** Immunological remission rates in patients with baseline aPLA2Rab ≤ 100 RU/mL. (*p < 0.05, ^**^p < 0.01, ^***^p < 0.001, ^****^p < 0.0001).

At baseline, all patients were positive for serum anti-PLA2R antibodies. By the 3-month follow-up, 9 patients had achieved seroconversion to antibody negativity, and by the end of the follow-up period, all patients had become seronegative, indicating complete immunological remission. Compared with baseline, anti-PLA2R antibody levels decreased significantly at 3, 6, and 12 months (all *p* < 0.001; Figure 3A), with mean reductions of 133.8 RU/mL, 173.0 RU/mL, and 179.6 RU/mL, respectively. The most pronounced decrease was observed at 12 months (95% CI: 96.82–262.3; *p* < 0.0001). When comparing antibody dynamics between high-risk and intermediate-low risk groups (Figure 3D), the high-risk group showed a slightly faster initial decline within the first 3 months. However, by the end of follow-up, both groups reached comparable seronegativity rates (*p* = 0.6, not statistically significant). The relative percentage reduction in anti-PLA2R antibody titers from baseline is illustrated in Figure 3B. Patients in the highest tertile (*n* = 5) maintained antibody levels > 25% of baseline at 4 weeks (suggesting a half-life > 7 days), yet only one remained positive by week 8, supporting the efficacy of obinutuzumab even in patients with high baseline titers. The overall immunological remission rates at 3, 6, 9, and 12 months were 70, 83, 90, and 93%, respectively, with complete immunological remission rates of 35, 74, 80, and 80% (Figure 3C). Among the 16 patients with baseline anti-PLA2R antibody > 100 RU/mL, complete immunological remission rates were 25, 56, 68, and 68% at the corresponding timepoints (Figures 3D,E). These results indicate that patients with lower baseline antibody titers achieved higher and faster remission rates, underscoring the early treatment response facilitated by obinutuzumab.

### 3.5 B-cells depletion kinetics

**FIGURE 4:**
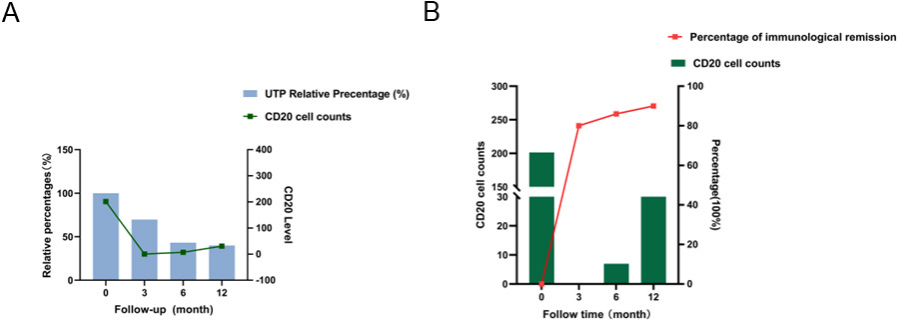
B-cell depletion kinetics. **(A)** Dual-axis plot of CD20^+^ cell count versus relative percentage change in 24-h urinary protein (UTP). **(B)** Dual-axis correlation between CD20^+^ cell count and immunological remission rate.

Obinutuzumab treatment induced rapid B-cell depletion, achieving complete depletion (CD20^+^ cells < 1 cell/μL) within the first 3 months. B-cell reconstitution began at month 6 and reached higher levels by month 12. Mean CD20^+^ cell counts at 0, 3, 6, and 12 months were 201, 0.2, 6, and 30 cells/μL, respectively. The relationship between B-cell depletion/reconstitution and changes in urinary total protein (UTP) is shown in Figure 4A. A sharp decline in UTP was observed concurrently with profound B-cell depletion, particularly after month 3. Notably, the onset of B-cell depletion preceded the reduction in proteinuria. During B-cell reconstitution (months 9–12), the rate of UTP decline slowed but remained on a stable downward trajectory, demonstrating a lag effect. By month 12, a mean reduction in UTP of 65% was achieved, indicating a favorable treatment response. Synchrony was observed between B-cell kinetics and immunological remission (Figure 4B). At month 3, 80% of patients had achieved immunological remission, coinciding with complete B-cell depletion. Although the rate of immunological remission decelerated during B-cell repopulation, a gradual improvement was sustained throughout follow-up.

Ten patients who had previously failed rituximab therapy were included in this study. Among them, four received a reduced per-infusion dose of obinutuzumab (0.6 g) for 3–4 cycles, while six received the standard per-infusion dose (1.0 g) for 2–5 cycles, yielding a median cumulative dose of 2.4 g (IQR: 2.0–3.0 g). Prior to obinutuzumab treatment, the mean baseline CD20^+^ B-cell count in these 10 patients was 168.33 cells/μL. After initiating obinutuzumab, all patients achieved complete B-cell depletion (mean < 5 cells/μL) by month 3. Although a trend toward B-cell repopulation was observed by month 6, the levels remained low.

### 3.6 Safety profile

Consistent with previously reported adverse effects of biologic agents (25), potential complications associated with obinutuzumab include infusion-related reactions (e.g., chills, fever, rash), infections, neutropenia, and hypogammaglobulinemia. However, in our observational cohort, obinutuzumab demonstrated a favorable safety profile. No patients progressed to end-stage renal disease or died during the follow-up period. Only 4 patients (12.9%) experienced mild and transient infusion-related reactions, including fever, hypotension, tachycardia, and facial flushing (Table 2). These symptoms did not recur during subsequent infusions. Although direct measurements are lacking in our results, based on the observed trends in B-cell depletion and reconstitution, changes in anti-PLA2R antibody titers, and urinary protein remission, we speculate that when most patients achieved clinical remission—following the clearance of pathogenic autoantibodies (anti-PLA2R antibodies)—partial recovery of immune system function occurred, accompanied by a gradual increase in IgG levels, while IgM levels remained stable or showed a slight decrease. This inference is consistent with previous clinical evidence regarding obinutuzumab or rituximab in the treatment of idiopathic membranous nephropathy (26, 27), which reports similar patterns in immunoglobulin dynamics during therapy.

**TABLE 2:** Adverse events observed in the study cohort.

Adverse event	Total (*n* = 31) n (%)
Fever	1
Hypotension	1
Tachycardia	1
Facial flushing	1

## 4 Discussion

This study evaluated the efficacy and safety of obinutuzumab monotherapy—without concurrent steroid pulses or other immunosuppressive agents—in patients with idiopathic refractory membranous nephropathy (IRMN), adhering strictly to the recommended obinutuzumab administration protocol. The treatment regimen was systematically and uniformly applied. In terms of clinical efficacy, the overall response rate (ORR) in this study cohort was 80%, with a complete remission (CR) rate of 16%. Notably, 45% of the refractory patients in this cohort had previously failed to respond to rituximab therapy, whereas historical reports indicate that rituximab achieves a response rate of approximately 68% in cohorts consisting mainly of treatment-naïve patients (28–30). This suggests that obinutuzumab is an effective treatment option for patients who respond inadequately to rituximab. Moreover, immunological responses occurred rapidly, with 41% of patients achieving remission as early as 1 month—preceding clinical remission—further supporting the potent B-cell depleting effect of obinutuzumab (31). Importantly, among the 9 patients who had previously failed rituximab therapy, 7 achieved remission after obinutuzumab treatment, representing a substantial clinical advance. Another key finding was the low relapse rate; only one case of relapse was observed during the study period. Although relapse rates have not been extensively analyzed in prior studies of obinutuzumab in MN, reported relapse rates for rituximab regimens are approximately 27% (16, 32). Our results suggest that obinutuzumab may significantly reduce the risk of disease relapse.

The complete remission (CR) rate in our cohort was 9% at 3 months and increased to 40% by 12 months. These results are consistent with those reported by Klomjit et al. (33), in which all 3 treated patients achieved immunological remission—2 with partial remission and 1 with CR (33% CR rate)—as well as with Sethi et al. (34), who observed a CR rate of 40% at a median follow-up of 6 months. Notably, our study included a substantially larger patient population than these earlier reports. Proteinuria level is a critical determinant of treatment response and prognosis in membranous nephropathy. In our study, the high-risk and intermediate-low risk groups exhibited reductions in 24-h UTP of 4.5 g/24 h and 3.79 g/24 h from baseline, respectively. Despite enrolling patients with refractory disease and high baseline proteinuria—factors typically associated with poorer outcomes—UTP levels decreased to below 50% of baseline by the third month of treatment. Serum albumin (ALB) levels also improved significantly during follow-up. Although most patients had normal serum creatinine (Scr) at baseline, those with elevated Scr at enrollment showed improved renal function by the end of the study, and the overall Scr level after treatment was lower than baseline. These findings collectively underscore the strong potential of obinutuzumab in the treatment of idiopathic refractory membranous nephropathy.

Regarding the safety of obinutuzumab in clinical use, we referred to the adverse event profile of rituximab, a first-generation anti-CD20 monoclonal antibody with a similar mechanism of action. The most frequently documented adverse event associated with such biologics is infusion-related reactions (35). Throughout the follow-up period, we cumulative monitored adverse events in our cohort. No severe adverse events were observed. Only a subset of patients experienced mild and transient reactions at the initiation of infusion, including fever, tachycardia, hypotension, and facial flushing.

Guided by the 2021 KDIGO guidelines, this study was deliberately confined to anti-PLA2R antibody-positive patients. This biomarker is pivotal not only for diagnosis but also for monitoring remission and predicting outcomes (36). Serial measurement of antibody levels offers an objective tool for evaluating immunological remission (37). By focusing on this specific subgroup and excluding non-PLA2R MN cases, we ensured a more homogeneous study cohort (38), thereby reducing confounding and strengthening the conclusions regarding treatment efficacy. However, this study has several limitations. It was a single-center, observational investigation with a limited sample size, which may introduce selection bias. Furthermore, the cohort was exclusively composed of anti-PLA2R antibody-positive patients. This selection was motivated by the pursuit of pathophysiological homogeneity and the availability of a quantifiable therapeutic monitoring metric (i.e., anti-PLA2R antibody titers), as this subgroup constitutes approximately 70–80% of IMN cases and represents an ideal model for investigating novel therapies. Consequently, the generalizability of our findings to PLA2R-seronegative patients (encompassing those with other autoantibodies or double seronegativity) may be limited. Future validation in larger, multi-center cohorts encompassing the full spectrum of IMN is therefore warranted.

## 5 Conclusion

The Obinutuzumab induced high remission rates in IRMN, with rapid immunological response and a tolerable safety profile, representing a novel therapeutic approach.

## Data Availability

The original contributions presented in this study are included in this article/supplementary material, further inquiries can be directed to the corresponding authors.
